# The diversity and clinical implications of genetic variants influencing clopidogrel bioactivation and response in the Emirati population

**DOI:** 10.1186/s40246-023-00568-3

**Published:** 2024-01-03

**Authors:** Lubna Q. Khasawneh, Habiba Alsafar, Hiba Alblooshi, Mushal Allam, George P. Patrinos, Bassam R. Ali

**Affiliations:** 1 Department of Genetics and Genomics, College of Medicine and Health Sciences, United Arab Emirates University, P.O. Box: 15551, Al-Ain, United Arab Emirates; 2 Department of Biomedical Engineering, Khalifa University of Science and Technology, Abu Dhabi, United Arab Emirates; 3 Center for Biotechnology, Khalifa University of Science and Technology, Abu Dhabi, United Arab Emirates; 4 ASPIRE Precision Medicine Research Institute Abu Dhabi, United Arab Emirates University, Al Ain, United Arab Emirates; 5 School of Health Sciences, Department of Pharmacy, Laboratory of Pharmacogenomics and Individualized Therapy, University of Patras, Patras, Greece; 6 Zayed Centre for Health Sciences, United Arab Emirates University, Al-Ain, United Arab Emirates

**Keywords:** Clopidogrel, Genetic variants, Pharmacogenomics, *CYP2C19*, *ABCB1*, *PON1*, *P2Y12R*, Emirati population

## Abstract

**Background:**

Clopidogrel is a widely prescribed prodrug that requires activation via specific pharmacogenes to exert its anti-platelet function. Genetic variations in the genes encoding its transporter, metabolizing enzymes, and target receptor lead to variability in its activation and platelet inhibition and, consequently, its efficacy. This variability increases the risk of secondary cardiovascular events, and therefore, some variations have been utilized as genetic biomarkers when prescribing clopidogrel.

**Methods:**

Our study examined clopidogrel-related genes *(CYP2C19, ABCB1, PON1,* and *P2Y12R)* in a cohort of 298 healthy Emiratis individuals. The study used whole exome sequencing (WES) data to comprehensively analyze pertinent variations of these genes, including their minor allele frequencies, haplotype distribution, and their resulting phenotypes.

**Results:**

Our data shows that approximately 37% (*n* = 119) of the cohort are likely to benefit from the use of alternative anti-platelet drugs due to their classification as intermediate or poor *CYP2C19* metabolizers. Additionally, more than 50% of the studied cohort exhibited variants in *ABCB1, PON1, and P2YR12* genes, potentially influencing clopidogrel’s transport, enzymatic clearance, and receptor performance.

**Conclusions:**

Recognizing these alleles and genotype frequencies may explain the clinical differences in medication response across different ethnicities and predict adverse events. Our findings underscore the need to consider genetic variations in prescribing clopidogrel, with potential implications for implementing personalized anti-platelet therapy among Emiratis based on their genetic profiles.

**Supplementary Information:**

The online version contains supplementary material available at 10.1186/s40246-023-00568-3.

## Background

Clopidogrel is a prodrug with anti-platelet properties that is frequently prescribed to prevent thromboembolic events in patients after the incidence of acute myocardial infarctions (MIs) [1], a relatively common and often lethal occurrence worldwide. When administered orally, clopidogrel requires intestinal absorption, activation in the liver, and binding to the extracellular receptor on platelets, inhibiting its function to exert its therapeutic action [2]. However, clopidogrel efficacy and response can differ significantly among individuals due, at least partly, to the presence of various genetic variants across its interacting proteins [3].

**Fig. 1:**
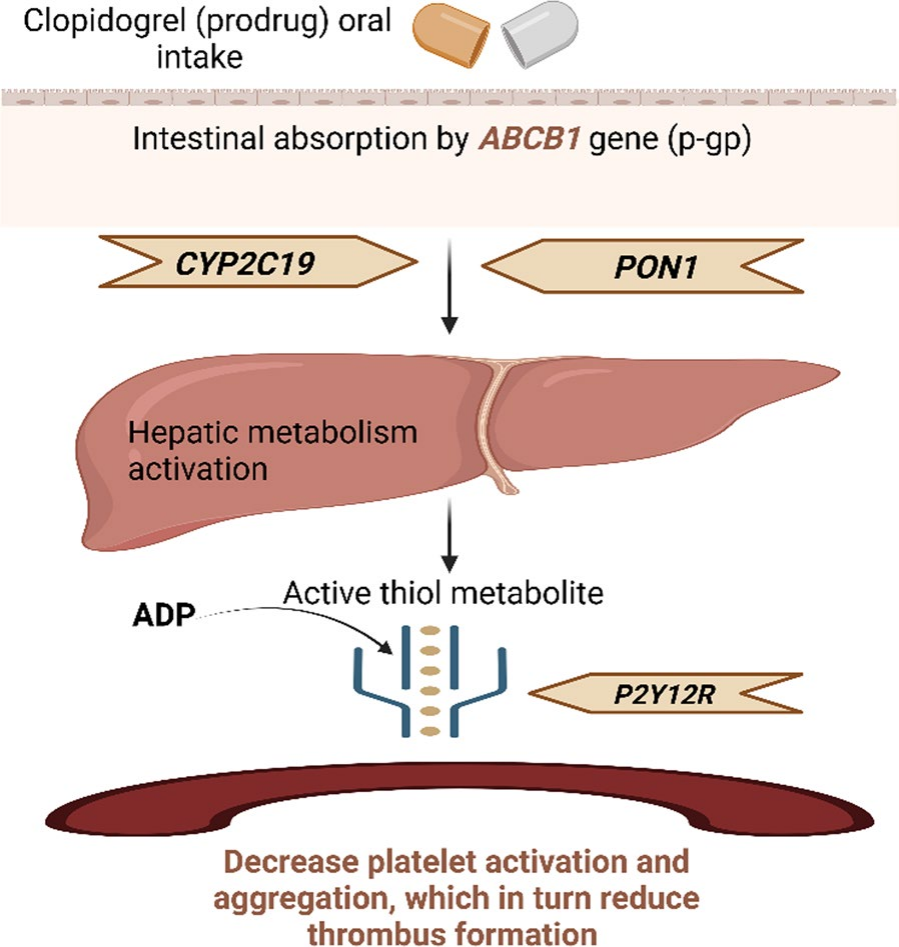
Pathway involved in clopidogrel transport, activation, and function. When clopidogrel is administered orally as a prodrug, the medication must be absorbed within the intestine, activated by two enzymatic steps in the liver, and finally bound to the platelets receptor to exert its biological action. The *ABCB1*gene plays a vital role in regulating the absorption of clopidogrel. Afterward, the metabolic oxidation process is facilitated via CYP2C19 and PON1 isoenzymes in the liver to form the active thiol metabolite, which must bind to the P2Y12R receptor to exert its bioactivity (diagram created by **BioRender**)

The contribution of the variants within the clopidogrel-related genes begins with limited oral bioavailability due to the intestinal absorption of the prodrug being mediated by the ATP-binding cassette subfamily B member 1 (encoded by *ABCB1*), a protein with variations that affect its transport function [4]. Once the medication has been absorbed, the hepatic activation occurs through metabolic oxidation v*ia* cytochrome P450 isoenzymes (predominantly CYP2C19) and non-P cytochromes through the hydrolytic cleavage of the thioester bond for the drug by Paraoxonase-1 (PON1) [5]. Subsequently, the active thiol metabolite should interact with the purinergic receptor (encoded by *P2Y12R*) on the platelet membrane [6] to exert its effects by decreasing platelet stimulation and subsequent coagulation. Thus, it reduces thrombus formation, which in turn decreases the risk of developing Major Adverse Cardiovascular Events (MACE), such as MIs, strokes, and others [7]. An illustration of the clopidogrel activation pathway and the major genes involved in this pathway is presented in Fig. 1.

The *ABCB1* gene, encoding p-glycoprotein (P-gp), is involved in the ATP-efflux drug transporter [8] and has a high degree of genetic diversity with more than 60 variants that have been studied in the past decade to determine their genetic-clinical impact*.* However, only three of those variants, namely rs104564 (C3435T), rs203258 (G2677T), and rs1128503 (C123T), have been suggested to restrict clopidogrel absorption through modulating its pharmacokinetic properties [9]. These three variants have been reported to be highly prevalent among various populations [10]. The full clinical impact of these variants is not well established and has not been fully investigated.

In addition, certain *CYP2C19* variants are among the earliest and most extensively studied in the pharmacogenetics (PGx) field and have been established as strong predictors of clopidogrel activation and response. For instance, the genetic variants rs4244285 (defining CYP2C19*2) and rs4986893 (defining CYP2C19*3) have been extensively documented as contributors to impaired drug metabolism status. These two loss-of-function (LoF) variants have been reported to diminish clopidogrel conversion to its active form, thereby confirming an increased risk of secondary cardiovascular events across multi-ethnic populations [11]. Conversely, CYP2C19*17 (defined by rs12248560), which is a gain-of-function (GoF) variant, exhibits increased enzymatic activity due to increased enzyme expression and acts as a protective factor by enhancing the platelet inhibition impact of clopidogrel [12].

Furthermore, two significant variants in the *PON1* gene at chromosome 7, namely rs662 (p.Q192R) and rs854560 (p.L55M), have been reported to affect clopidogrel metabolic activation in the liver by modulating the activity and stability of paraoxonase-1 enzyme. Research has shown that individuals carrying the minor R allele i.e., have glutamine Q amino acid replaced by arginine (R) in the rs662 variant (p.Q192R) tend to have higher paraoxonase-1 activity in comparison with those who have the common Q allele [13]. In contrast, the substitution of Leucine (L) amino acid with methionine (M) in the rs854560 (p.L55M) variant has been associated with a reduction in the stability of paraoxonase protein, leading to a decrease in the serum concentration of the enzyme itself [14]. Both variants have been confirmed to have a role in cardiovascular pathogenesis [15].

The *P2Y12R* gene, also known as the purinergic receptor P2Y, is predominantly expressed on platelets and codes for G-protein signaling receptors that specifically react with the extracellular purine, namely adenosine diphosphate (ADP). As clopidogrel is a P2Y12R inhibitor that blocks the ADP-mediated signaling pathway, five variants (rs10935838, rs2046934, rs5853517, rs6785930, and rs6809699) in this gene have shown association with decreased inhibition of platelets aggregation accelerating the atherosclerosis process and consequently clot formation and eventually lead to a higher risk of cardiovascular complications in patients taking clopidogrel [16].

The PGx research concerning clopidogrel in the United Arab Emirates (UAE) is in its nascent stage and has primarily been underexplored [17, 18]. Hence, there is a pressing need to delve into the genetic factors influencing clopidogrel response within the local population for implementing effective and personalized anti-platelet treatments. Therefore, in this study, we examined the allele and genotype frequencies for genetic variants in the four genes (*ABCB1, CYP2C19, PON1,* and *P2Y12R*) that affect the conversion of clopidogrel into the biologically active metabolite in a sample of 298 healthy individuals from the Emirati population, utilizing whole exomic data. Additionally, we compared the observed alleles and genotype frequencies with those identified in other populations. The study also included the presentation of an extensive summary of the existing knowledge regarding the haplotype analysis, diversity, and frequency of the variants linked to clopidogrel response.

## Methods

### Study participants

Two hundred and ninety-eight (*n* = 298) healthy adult volunteers from the United Arab Emirates national population participated in the study. Each participant provided blood samples and signed informed consent forms. The study has been approved by the ethical committee within the Department of Health/Abu-Dhabi, bearing the approval code DOH/ CVDC/2022/1450, for individuals involved in the project recruited at the United Arab Emirates University (UAEU), Al-Ain, United Arab Emirates. While for the participants from Abu-Dhabi and Dubai, recruited at Khalifa University (KU), Abu-Dhabi, were approved by the ethical committee of Dubai Scientific Research Ethics Committee, under the identification code DSREC-07/2020_19, A-2021-070.

### Samples processing

Genomic DNA was isolated from whole blood samples that had been collected in EDTA tubes, employing either the automated MagPurix Blood DNA Extraction Kit 200 (Zinexts, New Taipei City, Taiwan) or the manual QIAamp DNA Blood Kits kit (Qiagen, Hilden, Germany). These extractions were carried out in accordance with the manufacturer’s protocols. Subsequently, the quantification of the DNA was performed utilizing the DS-11 Series Spectrophotometer/Fluorometer (DeNovix, Wilmington, Delware, USA) or the Nanodrop One Spectrophotometer (Thermo Fisher Scientific, USA). The DNA samples were then analyzed using Whole Exome Sequencing (WES). For KU samples, the subsequent library sequencing was conducted using the Illumina NextSeq 500/550 system (Illumina, Inc., San Diego, California, USA). The NSQ 500/550 High Output kit v2.5 for 150 cycles of sequencing (Illumina, Inc., San Diego, California, USA) was performed to achieve the desired sequencing parameters. While for UAEU samples, library’s concentration and fragment size were determined using the Agilent 4200 TapeStation system (HS D1000 ScreenTape Assay; Agilent Technologies, USA). The final normalized libraries were sequenced using SP and S1 flow cell on NovaSeq 6000 platform (Illumina, Inc., San Diago, California, USA).

Primary and secondary analysis of the raw data generated by the sequencing machines from both sites was carried out using the Illumina DRAGEN BIo-IT 4.00 (Illumina, Inc., San Diego, California, USA). Using this platform, we efficiently extracted, filtered, and annotated the Variant Calling Format (VCF) files, with focus on specified genes of interest.

For the purpose of this study, we focused our analysis on 11 single nucleotide polymorphisms (SNPs) from the four genes *(CYP2C19, ABCB1, PON1,* and *P2Y12R*) that affect clopidogrel activation, metabolism, and action. A list of the assessed SNPs and their reported potential clinical effects on clopidogrel efficacy and toxicity are summarized in Table 1.

**Table 1:** The list and characteristics of variants analyzed in the current Emirati cohort

Gene	SNP ID & sub	Synonym	Type	Potential clinical effect*	Clinical annotation Level of evidence (PharmGKB)	References
*CYP2C19*	rs4244285 (c.681G > A)	CYP2C19*2	Synonymous Splice variant	Decrease bioavailability (LoF variant). Increase MACE	Level 1A	[11, 19]
*CYP2C19*	rs4986893 (c.636G > A)	CYP2C19*3	Stop gained	Decrease bioavailability (LoF variant). Increase MACE	Level 1A	[19, 20]
*CYP2C19*	rs12248560 (c.-806C > T)	CYP2C19*17	Intronic variant on the 5’ flanking region	Increase bioavailability (GoF variant)	Level 1A	[19, 21]
*CYP2C19*	rs12769205 (12662A > G)	CYP2C19*2/*35	Synonymous splice variant	Decrease bioavailability. Increase MACE	Level 1A	[19]
*ABCB1*	rs1045642 (c.3435 T > C)	C3435T	synonymous variant	Decrease bioavailability, drug response, Increase MACE	Level 3	[22]
*ABCB1*	rs2032582 (c.2677G > T)	G2677T	Missense variant	Decrease bioavailability, drug response, Increase MACE	Level 3	[23]
*ABCB1*	rs1128503 (c.C123)	C123T	Missense variant	Decrease bioavailability, drug response, Increase MACE	Level 3	[23]
*PON1*	rs662 (c.575A > G)	Q192R	Missense variant	Increase paraoxonase activity. Increase MACE	Level 4	[24]
*PON1*	rs854560	L55M	Missense variant	Decrease the serum concentration of paraoxonase enzyme	Level 4	[25]
*P2Y12R*	rs6785930 (c.18C > T)	C34T	Missense variant	Reduce response to clopidogrel. Increase MACE	Level 4	[26]
*P2Y12R*	rs6809699	G52T	Synonymous variant	Increase clopidogrel resistance	Level 3	[27]

*Potential clinical effect based upon PharmGKB database

SNP, Single Nucleotide Polymorphism; CYP, Cytochrome P; *ABCB1*, ATP-binding Cassette Subfamily B Member 1; LoF, Loss Of Function; GoF, Gain Of Function; MACE, Major Adverse Cardiovascular Events; *PON1*, Paraoxanase 1; *P2Y12R*, Purnegic PY12 Receptor

### Haplotypes annotation, genotype–phenotype analysis, and correlations

The studied genotypes for clopidogrel-related variants were translated into haplotypes, diplotypes, and to the predicted phenotypes using the Pharmacogene Variation Consortium (PharamVar) (PharmVar), Clinical Implementation Pharmacogenomics Consortium (CIPC) guidelines (Guidelines–CPIC (cpicpgx.org)), and the Pharmacogenomics Knowledge Database (PharmGKB). PharmVar was used to extract *CYP2C19* star alleles, while for all other alleles in *ABCB1, PON1,* and *P2RY12* genes, PharmGKB provided comprehensive information about the effect of these genetic variants on clopidogrel response. Additional information regarding the translation of the haplotypes and dipoltypes into phenotypes can be found in the accompanying Additional file 2.

### Statistical analysis

The Hardy–Weinberg equilibrium test was assessed using the chi-square (*x*^2^) test based on the following equation P2 + 2pq + q2 = 1. GraphPad Prism version 9.5.1 was used for the chi-square test to compare the study’s genetic variants’ allele and genotype frequencies. A *p* value lower than 0.05 was considered statistically valid.

## Results

### The frequencies of the clopidogrel-related variants among the Emirati cohort are in Hardy–Weinberg Equilibrium (HWE)

The distribution of the minor allele frequency (MAF) of variants in clopidogrel-relevant genes among the 298 Emirati participants is presented in Table 2. All the alleles were in Hardy–Weinberg Equilibrium, with a *p* value larger than 0.05, indicating that the selected group is representative of the larger Emirati population for studying the genetic variants associated with clopidogrel metabolism (i.e., the observed allele frequencies for all tested SNPs are consistent with expected frequencies under the genetic disequilibrium).

**Table 2:** MAF distribution among the current Emirati cohort

Genetic variants	MAF (%)	*P* value	*X*^2^
*CYP2C19*			
rs4244285 (CYP2C19*2)	19.3	0.054	3.78
rs4986893 (CYP2C19*3)	0.4	0.558	0.841
rs1224856(CYP2C19*17)	19.7	0.963	232.05
rs12769205 (CYP2C19 *2/*35)	20.5	0.558	0.345
*ABCB1*			
rs1045642	55.9	0.154	7.327
rs2032582	55.5	0.257	7.664
rs1128503	55.46	0.374	9.899
*PON1*			
rs662 (Q192R)	38.6	0.088	34.502
rs854560 (L55M)	32.03	0.382	77.641
*P2Y12R*			
rs6785930 (C34T)	27.12	0.551	92.199
rs6809699 (G52T)	95.94	0.4861	420.716

MAF, minor allele frequency

It has been observed (in Table 2) that the CYP2C19*3 star allele had the lowest prevalence among Emiratis, accounting for only 0.4% of the *CYP2C19* alleles at the defining position. On the other hand, the rs680699 (G52T) variant in *P2Y12R* showed a high prevalence of 95.94% of the Emirati cohort carrying at least one minor allele of this particular variant.

### Comparisons of the minor allele frequencies (MAF) of the studied variants with other populations

**Fig. 2:**
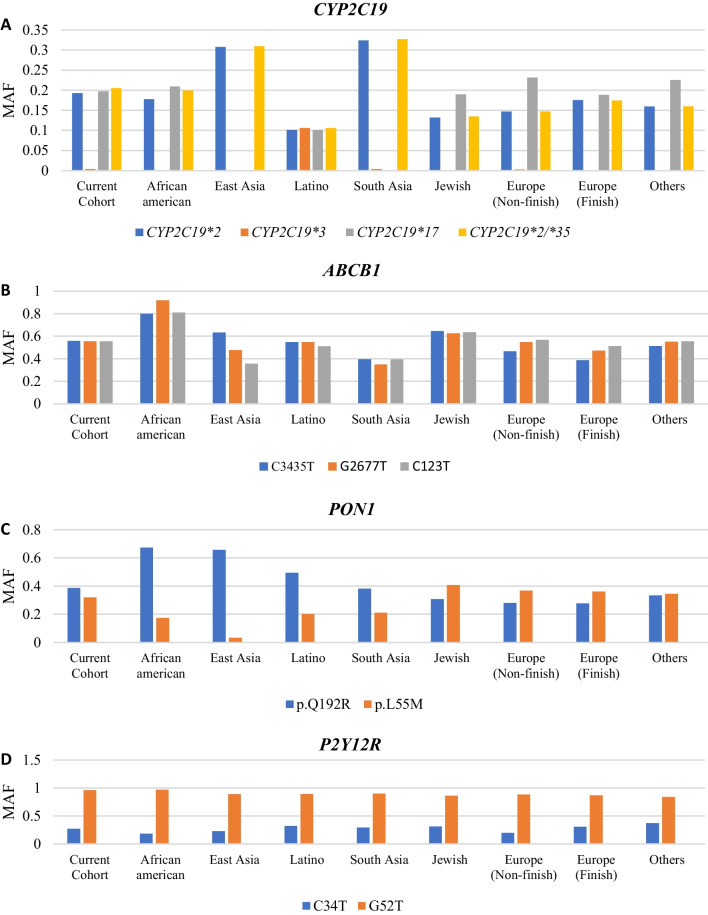
Minor allele frequency (MAF) distribution between different populations for clopidogrel-related genes

The Minor Allele Frequency (MAF) from other populations was obtained from the Genome Aggregation Database (GenomAD) (gnomAD (broadinstitute.org)), which offers comprehensive data on allele count and numbers for eight subpopulations, including African American (AF), East Asia (EA), Latino, South Asia (SA), Ashkenazi Jewish, European Finish and non-Finish, and others. The MAF comparison between the current Emirati cohort and the eight subpopulations for all the examined variants from the four genes are presented in Additional file 1: Table S1 and summarized in Fig. 2.

For the variants associated with the *CYP2C19* in Fig. 2A, the MAF for the two splicing variants defining the CYP2C19*2 allele, specifically rs4244285 and rs12769205, were similar across all the ethnic groups due to the high Linkage disequilibrium (LD) between them. In addition, the CYP2C19*17 allele had a relatively consistent MAF across most populations, with the exception of those from China, Japan, the Philippines, Korea, and other parts of East Asia, where the MAF was nearly zero. For the CYP2C19*3 allele, the MAF is generally low in most populations, except for the Latino population, where it exhibited a relatively high frequency of 0.1058.

The *ABCB1*-associated variants, specifically rs1045642, rs2032582, and rs1128503, exhibited a relatively high MAF in the Emirati cohort, as presented in Fig. 2B (0.559, 0.555, and 55.46, respectively), which is similar to most other populations such as East Asia, South Asia, and Latinos. Yet, these frequencies are lower than those of African Americans (0.799, 0.917, and 0.811, respectively).

For the MAF displayed for the two *PON1* variants in Fig. 2C, our cohort revealed that both Q192R and L55M variants had relatively comparable frequencies (i.e., MAF values of 0.386 and 0.321, respectively) contradicting the differences observed in African American, Latino, and East Asia populations.

The MAF was highest for the *P2RY12* rs6809699 (G52T) variant, observed at a value of 0.959 to all other variants tested in our Emirati cohort. When comparing the same variant with other ethnic groups, African Americans had a similarly high minor allele frequency (0.9683). Conversely, another *P2Y12R* variant, rs6785930 (C34T), was observed to have higher MAF than African Americans, European non-Finish, and East Asian groups (with an MAF value of 0.2712) But lower than the rest of the other populations (such as Jewish, south Asia, and Finnish Europeans populations).

### Prevalence of *ABCB1* and *CYP2C19* genes haplotypes/diplotypes with the predicted phenotypes in the Emirati cohort

**Fig. 3:**
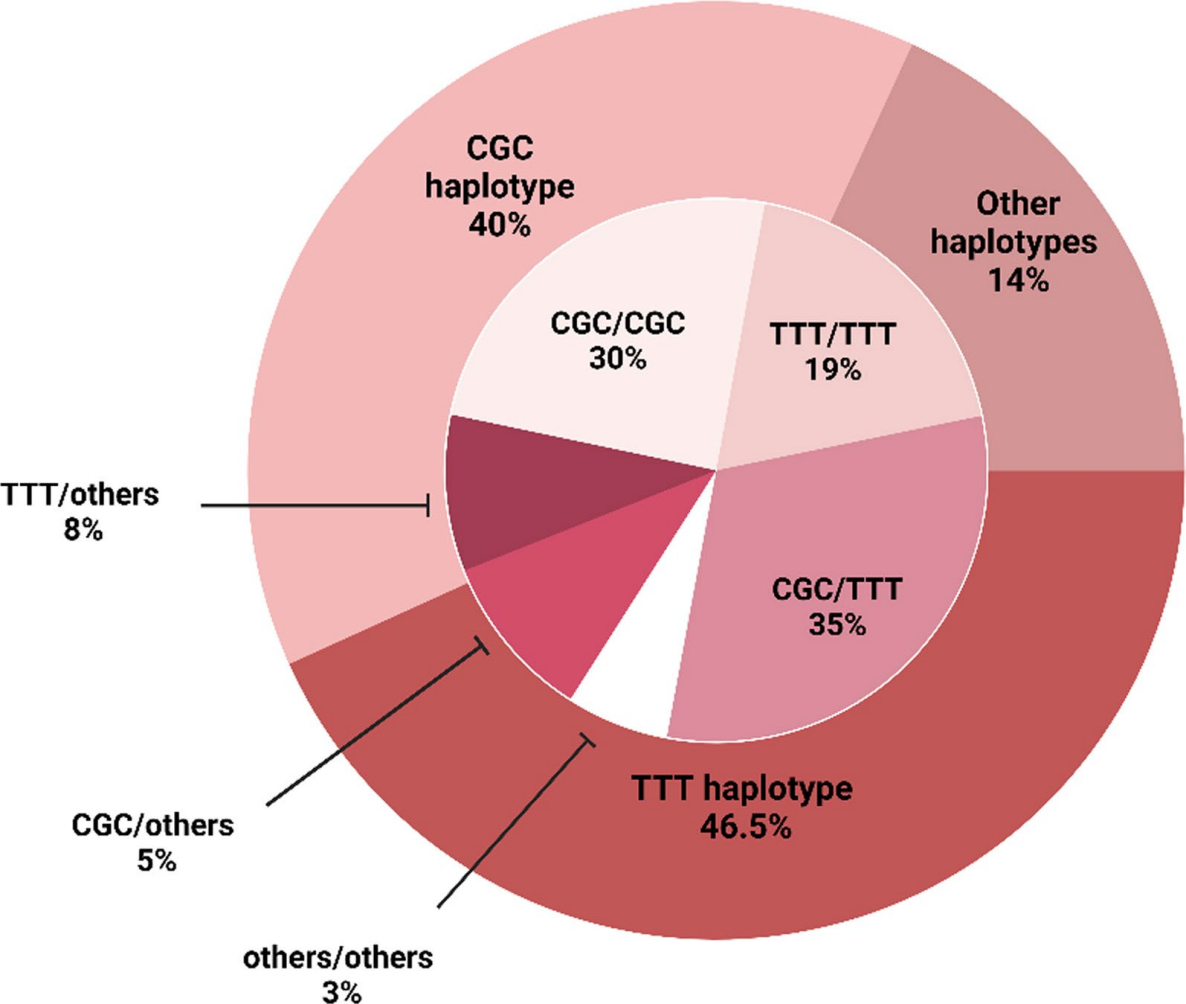
*ABCB1* haplotypes and diplotypes distribution among the current Emirati cohort

As shown in in Fig. 3, the annotation of haplotypes for the three *ABCB1* variants revealed that approximately 40% (*n* = 113) of participants carried the reference allele for all the variants (C3435-G2677-C123 haplotype). In comparison, around 46.5% harbored the mutant variants (3435T-2677T-123T haplotype). When combining the *ABCB1* haplotypes to predict the impact on the genes’ pharmacokinetic profile, it was found that 19% of the studied cohort carried two mutant alleles from all variants (TTT/TTT). In contrast, nearly 35% of participants had only the TTT mutant haplotype (i.e., predicated diplotypes were TTT/CGC). Yet, about one-third of participants (30%) predicted having normal ABCB1 enzymatic activity since they carry two reference haplotypes (CGC/CGC).

**Fig. 4:**
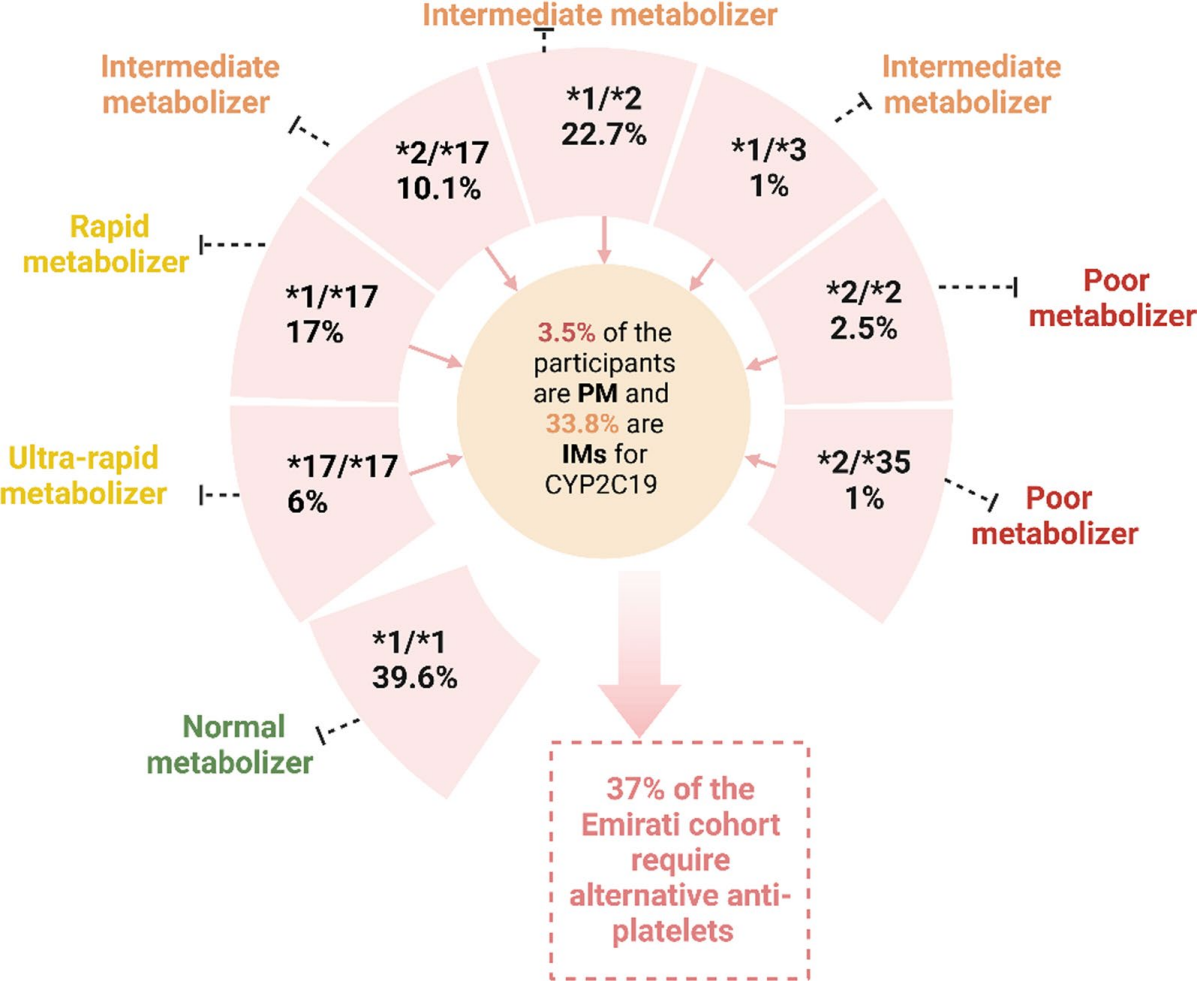
CYP2C19 predicted metabolizing status (Created by **BioRender**)

As for the predicted CYP2C19 metabolizing status illustrated in Fig. 4, the study revealed that approximately 40% of our sample exhibited a predicted regular metabolic activity (i.e., normal metabolizers ((NM)), 37% classified as either intermediate or poor metabolizers ( i.e., those with two non-function alleles resulting in no enzymatic activity), and 23% fell into the category of rapid (RM) or ultra-rapid metabolizers (URM) (i.e., those with an accelerated rate of CYP2C19 enzyme metabolism*)*. As per the *CYP2C19*-clopidogrel guidelines offered by the CPIC consortium with the recently updated recommendations [19], the metabolizing status for IM and PMs individuals carries clinical decision-making implications regarding clopidogrel therapy. These guidelines advocate for substituting clopidogrel with alternative anti-platelet medications such as prasugrel or ticagrelor in patients identified as IM or PMs. Thus, 37% of the Emirati cohort, when they experience an incidence of myocardial infractions, should be prescribed other anti-platelets rather than clopidogrel to minimize the risk of recurrent cardiovascular attacks and improve their health outcomes.

### Prevalence of *PON1* variants, combined genotypes, and the predicted phenotypes in the Emirati cohort

**Fig. 5:**
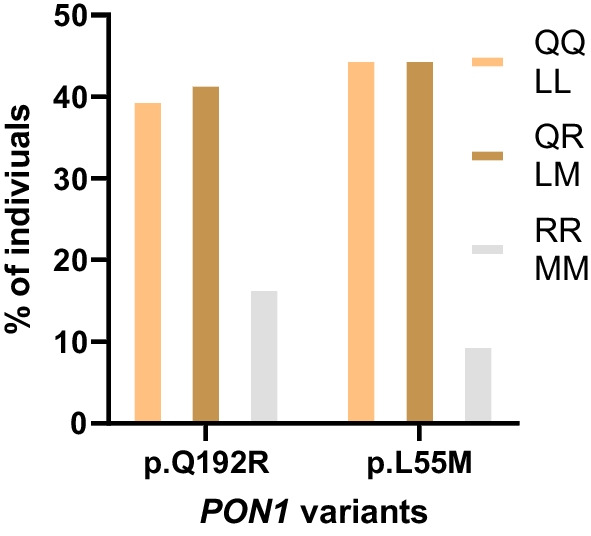
Distribution of p.Q192R and p.L55M variants genotype among the Emirati cohort

Figure 5 shows the prevalence of *PON1* variants among the studied Emirati cohort. It is worth highlighting that approximately 60% of the cohort is anticipated to have reduced serum paraoxonase activity of the PON1 enzyme, attributed to the presence of one or two minor alleles from the p.Q192R variant (i.e., QR or RR genotype). In contrast, the remaining 40% of the cohort is projected to maintain a high serum concentration of the enzyme, denoting regular PON1 enzymatic activity (i.e., QQ genotype). Regarding the p.L55M variant, similar percentages with 55% of the participants are expected to exhibit destabilization of the PON1 enzyme due to the presence of at least one mutant allele in the p.L55M variant (i.e., LM or MM genotype), leading to a notable decrease in its serum concentrations. On the other hand, the rest of the study cohort (approximately 35%) maintained a stabilized PON1 activity by harboring both reference alleles from the p.L55M variant (i.e., LL genotype).

For the evaluation of the combined genotype distribution for the two *PON1* variants, the findings are displayed in Table 3. Only 34 individuals in our cohort (11.41%) are predicted to have a high PON1 enzymatic activity (i.e., participants bearing both reference alleles for the two *PON1* variants (QQ and LL). Intriguingly, the majority of the cohort, 264 out of 298 (88.59%), possessed at least one alternative allele of one of the two variants (rs662 and rs854560). Specifically, of these 264 participants with alternative alleles, 69 individuals were identified as heterozygous for both *PON1* variants. These variant alleles were reported to negatively affect either the stability of the PON1 enzyme or its serum concentrations [20].

**Table 3:** Combined genotype data for PON1 variants in the current Emirati group

*PON1* variants	rs854560 (p.L55M)		
rs662 (p.Q192R)	LL	LM	MM
QQ	34 (11.41%)	61 (20.47%)	25 (8.39%)
QR	57 (19.13%)	69 (23.15%)	1 (0.34%)
RR	44 (14.77%)	6 (2.01%)	1 (0.34%)
Total	135	136	27

The *P* value was < 0.00001

## Discussion

The variability in the effectiveness and response to clopidogrel anti-platelet therapy is widely recognized worldwide but not yet within the UAE local population. In our investigation, we report the frequencies of the genetic determinants impacting clopidogrel metabolism and response within a pooled sample from the Emirati population, which may offer an understanding of the specific differences in clopidogrel response observed within this population.

### Influence of *CYP2C19* and *ABCB1* genetic variants

As expected with a gene exhibiting a high degree of polymorphism like *CYP2C19*, PharmGKB has pinpointed a “must-test” array of *CYP2C19* variants, encompassing CYP2C19 *2, CYP2C19*3 and CYP2C19*17. These are recognized as tier 1 variants in clinical and population-specific investigations due to their proven impact on interindividual variability in drug responses [21]. The frequency of the CYP2C19 metabolizing status demonstrates a broad range across worldwide populations [22]. The analysis of CYP2C19*2 in a prior study involving 100 healthy Emirates revealed a lower MAF with a value of 0.151 [17]. In contrast, the current cohort exhibited a slightly higher MAF at 0.193. For CYP2C19 metabolizing status, 3.5% of the studied Emiratis displayed a significant decline in *CYP2C19* enzymatic activity, thus being classified as poor metabolizers (PM). The incidence of PMs in this cohort exceeded that observed in Caucasians (approximately 2%) but fell short of what was reported for other populations [23]. Furthermore, one-third of the participants were IMs for *CYP2C19*, aligning with the prevalence seen in the South Asian group, yet lesser than the prevalence reported in the East Asians, where IM individuals comprised over 45% of the population. Thus, 37% of the participants (i.e., both IM and PMs) are at higher risk of developing MACE events and require urgent PGx intervention based on the published CPIC recommendations. These results imply that preemptive PGx testing could be beneficial in predicting the side effects of clopidogrel and assist physicians in avoiding potential adverse effects by prescribing alternative anti-platelet medications based on the individual’s genetic testing results. Acknowledging the significance of PGx intervention, practical implementation feasibility might face various hurdles for those patients [24, 25]. One hurdle is the need to enhance awareness among healthcare professionals and stakeholders regarding the crucial integration of PGx into routine clinical practice [26–28]. Additionally, financial strategies should be explored to address the higher cost of ticagrelor compared to clopidogrel. This may involve seeking government support and advocating for insurance reimbursement to make PGx more economically viable in the healthcare settings [29, 30].

The genetic assessment for *ABCB1* gene haplotypes in the current study further corroborates the frequency observed in other populations [31–33]. The CGC and TTT haplotypes presented the highest percentages, accounting for 40% and 46.5%, respectively, reflecting the prevalence is due to the robust LD among the three prevalent *ABCB1* variants. However, the literature indicates the absence of standardization regarding the translation of haplotypes into defined star allele designation and phenotypic prediction. To address this, we refer to the star allele designation that was proposed by Kim and coworkers [31]. In accordance with this designation, the C3435-G2667-C123 haplotype was denoted as the reference *1 allele, whereas the 3435T-2667T-123T haplotype was designated as *2 (mutant allele). Consequently, the combinations CGC/CGC, CGC/TTT, and TTT/TTT can be retrospectively translated as *1/*1, *1/*2, and *2/*2 based on the star allele nomenclature. Hence, based on the results displayed in Fig. 3, nearly 19% of the cohort was found to carry the mutant *2/*2, and around 35% had the *1/*2 diplotype. Overall, these findings indicate that approximately 54% of the cohort, comprising carriers of either the *1/*2 and *2/*2 genotypes, may experience substantial variability in the pharmacokinetics profile for clopidogrel response. But the clinical impact of these haplotypes is still underestimated [34]. Indeed, further studies are needed to better define the star allele designation and its phenotypic consequences in different ethnicities among clopidogrel users.

Clinical investigations have concluded no direct interaction between *CYP2C19* loss of function variants and *ABCB1* variants [35]. Nevertheless, it has been documented that individuals carrying genetic variants in both *CYP2C19 and ABCB1* genes have been associated with a fivefold surge in cardiovascular complications compared to those having the reference alleles from both genes. Thus, the concurrent presence of *CYP2C19 and ABCB1* variants in individuals contributes additively to elevating the incidence of subsequent cardiovascular events [36]. Hence, a deeper investigation of the cumulative effect of the presence of both *ABCB1* and *CYP2C19* variants in the Emirati population is essential.

### Influence of *PON1* and* P2Y12R* genetic variants

The paraoxonase enzyme, which is involved in the metabolic activation of clopidogrel, plays a critical role in the *PON1* gene variant, specifically the p.Q192R. The p.Q152R were found to have high MAFs in the current investigation, which was aligned with observations in other Arab regions, including Jordan (28.7%) [37] and Saudi Arabia (36%) [38]. However, it was less than that seen in East Asian nations such as Japan (60%) [39] and China (53.7%) [40]. Regarding the p.L55M variant of the *PON1* variant, which modulates the paraoxonase enzyme serum concentrations, the existing MAF in the Emirati population was reported to be higher than that reported in East Asian countries [41]. In addition, analysis of the frequencies of the combined genotypes displayed in Table 4 showed that they were consistent with all frequencies presented by Janicsek and coworkers [41].

Since several studies have explored the positive association between these two *PON1* variants and the susceptibility to coronary artery diseases [42]. When assessing the combined effect of these two variants, researchers can investigate potential interactions or synergistic effects that may impact paraoxonase enzymatic activity and, in turn, better understand the functional phenotypic consequences related to cardiovascular outcomes. In the context of clopidogrel response, further research is warranted to fully elucidate the effect of the combined *PON1* variants on the PON1 function, particularly concerning clopidogrel efficacy to minimize the cardiovascular consequences in patients prescribed clopidogrel.

Regarding variants located on the P2-mediated receptor platelet 12 (*P2Y12R*) gene, our study substantiates significant genetic heterogeneity in the rs6809699 (G52T) variant and a relatively marked minor allele prevalence in the other variant, namely rs6785930 (C34T). These findings suggest that these genetic variations may influence the individual risk of developing ischemic incidents by increasing clopidogrel resistance through modulating platelet activation and aggregation [43]. The positive association between the two *P2Y12R* variants separately and ischemic events was previously confirmed by several PGx studies conducted on patients who received clopidogrel after heart attacks and were found to have a genetic variant in the *P2Y12R* variants [44].

Lastly, it is crucial to underscore that the clinical implication and utility of employing *ABCB1*, *PON1,* and *P2Y12R* variants in directing clopidogrel treatment remain unconfirmed as they are still classified under levels three and four. Further comprehension of the independent contribution of each variant to clopidogrel efficacy and safety will aid in including these variants in the tested list in PGx panels. This inclusion will broaden the scope of PGx testing for clopidogrel in different clinical settings.

### Limitations

Our study is subject to several limitations. Firstly, given that this study was conducted on healthy Emirati individuals, the connection between some clopidogrel-relevant genes and the clinical consequences in patients’ post-acute myocardial events may not be fully understood. Therefore, to address this limitation and to enhance the clinical importance of our findings, further research on the impact of these variants on the active metabolite concentrations, coupled with end clinical outcomes in actual patients treated with clopidogrel, is necessary. By studying a broader patient population, we can better assess the relevance of the identified genetic variants in real-world clinical scenarios and their impact on treatment response and cardiovascular outcomes.

Secondly, the WES coverage was not enough to cover three promotor variants modulating the *P2RY12* gene, specifically rs10935838, rs2046934, and rs5853517. A comprehensive analysis of the *P2RY12* genes’ genetic variants and their influence on clopidogrel bioactivation could be achieved by examining all five SNPs, including rs6785930 and rs6809699 from the current study. This would pave the way for an in-depth understanding of the haplotypes associated with this gene and their significance in determining individual responses to the drug. It is advisable to conduct additional studies with larger sample sizes considering environmental and lifestyle factors to yield more precise and robust findings. These studies could offer a more comprehensive understanding of the genetic diversity within the UAE population concerning the causes of hypo-responsiveness to clopidogrel.

## Conclusions

Clopidogrel stands as the most frequently prescribed anti-platelet medication. Establishing the allele and genotype frequencies for various clopidogrel-related genes can pave the way for PGx studies tailored to diverse populations. These comprehensive and inclusive PGx population studies serve as a road map, ensuring the precision and applicability of PGx findings, particularly in populations that have been understudied. Emphasizing such studies are essential to facilitate the transition of variants from levels three and four to level one with clinical recommendations, assisting in the inclusion of these variants in preemptive PGx testing panels in patients prescribed clopidogrel worldwide. Moving forward, it is crucial to recognize the broader implications of our study by bridging the gap between genetic findings and real-world patient care. This advancement would significantly contribute to tailoring personalized medicine strategies by integrating PGx data into routine clinical practice. Ultimately, this integration has the potential to transform the landscape of clopidogrel therapy for the betterment of individuals’ lives, based on the unique genetic profiles worldwide.

### Supplementary Information

**Additional file 1**. **Table S1** Additional representations for distributing the Minor allele frequency among different populations for clopidogrel-related genes.

**Additional file 2**. Additional information on translating genotypes into haplotypes, diplotypes, and predicted phenotypes.

## Data Availability

The datasets used and analyzed during the current study are not publicly available due to the regulations of the local ethical committee but are available from the corresponding author upon reasonable request.
