# Supplementary material for: The diversity and clinical implications of genetic variants influencing clopidogrel bioactivation and response in the Emirati population
Source: Hum Genomics. 2024 Jan 3;18:2. doi: 10.1186/s40246-023-00568-3 (PMC10765826; doi:10.1186/s40246-023-00568-3)
Supplement: Supplementary file 1 — Additional file 1. Table S1 Additional representations for distributing the Minor allele frequency among different populations for clopidogrel-related genes. [file 40246_2023_568_MOESM1_ESM.docx]

**Additional file 1. Supplementary Information**

**Table S1:** the distribution of Minor allele frequency for clopidogrel-related genes among different populations.

| **Genes /variants** | Current cohort | African American  (AF) | East Asia (EA) | Latino | South Asia (SA) | Jewish | Europe (non-finish) | Europe (Finish) | Others |
| --- | --- | --- | --- | --- | --- | --- | --- | --- | --- |
| ***CYP2C19*** |  |  |  |  |  |  |  |  |  |
| rs4244285  *CYP2C19*2* | 0.193 | 0.1776 | 0.3075 | 0.1012 | 0.324 | 0.132 | 0.1468 | 0.175 | 0.1595 |
| rs4986893  *CYP2C19*3* | 0.004 | 0.00036 | 0.000198 | 0.1058 | 0.004 | 0 | 0.0026 | 0.0008 | 0.0002 |
| rs12248560  *CYP2C19*17* | 0.197 | 0.2092 | 0.0007 | 0.1007 | 0 | 0.1897 | 0.2314 | 0.1884 | 0.2255 |
| rs12769205  *CYP2C19 *2/*35* | 0.205 | 0.199 | 0.3095 | 0.1058 | 0.3272 | 0.1344 | 0.1467 | 0.174 | 0.16 |
| ***ABCB1*** |  |  |  |  |  |  |  |  |  |
| rs1045642  C3435T | 0.559 | 0.7993 | 0.6321 | 0.548 | 0.3952 | 0.6441 | 0.4663 | 0.386 | 0.5127 |
| rs2032582  G2677T | 0.555 | 0.9167 | 0.4765 | 0.5476 | 0.3494 | 0.6247 | 0.5476 | 0.4699 | 0.5512 |
| rs1128503  C123T | 0.5546 | 0.8106 | 0.3564 | 0.5111 | 0.3933 | 0.6335 | 0.5671 | 0.513 | 0.555 |
| ***PON1*** |  |  |  |  |  |  |  |  |  |
| rs662  p.Q192R | 0.386 | **0.6733** | o.6568 | o.4941 | 0.3824 | 0.3075 | 0.2806 | 0.2771 | 0.3343 |
| rs854560  p.L55M | 0.3203 | 0.1728 | 0.03413 | 0.2012 | 0.21 | 0.4077 | 0.367 | 0.3607 | 0.3457 |
| ***P2Y12R*** |  |  |  |  |  |  |  |  |  |
| rs6785930 | 0.2712 | 0.1814 | 0.2279 | 0.321 | 0.2938 | 0.3098 | 0.1958 | 0.3078 | 0.3708 |
| rs6809699 | 0.9594 | 0.9683 | 0.889 | 0.8931 | 0.9013 | 0.8593 | 0.8836 | 0.8681 | 0.8405 |
