# Supplementary material for: The diversity and clinical implications of genetic variants influencing clopidogrel bioactivation and response in the Emirati population
Source: Hum Genomics. 2024 Jan 3;18:2. doi: 10.1186/s40246-023-00568-3 (PMC10765826; doi:10.1186/s40246-023-00568-3)
Supplement: Supplementary file 2 — Additional file 2. Additional information on translating genotypes into haplotypes, diplotypes, and predicted phenotypes. [file 40246_2023_568_MOESM2_ESM.docx]

**Supplementary file S2: Additional information on translating genotypes into haplotypes, diplotypes, and predicted phenotypes**

Based on PharmGKB, the analysis of *CYP2C19* genotypes is classified into five phenotypes: (1) Normal Metabolizers (NM) are individuals who are homozygous for the normal function allele (i.e., *1/*1), (2) Intermediate Metabolizer (IM) individuals carrying one LOF alleles ( i.e., one-star allele *2 or *3), which is translated into (either *1/*2 or *1/*3), (3) Poor Metabolizer (PM) individuals are homozygous for loss of function alleles (i.e., carrying two LOF alleles), which interpreted as *2/*2, *2/ *3, or *3/*3, (4) Rapid Metabolizers (RM) individuals are heterozygous for the gain of function *17 star allele (i.e., *1/*17) and (5) Ultra-Rapid Metabolizers (URM) individuals for those having two gain of function alleles (*17/*17).

In the context of the *ABCB1* gene haplotypes, it has been indicated that the three *ABCB1* variants (C3435T, G2677T, and C123T) are closely positioned and segregated together within the *ABCB1* gene sequence. Due to the high linkage disequilibrium (LD) pattern observed between them, the extracted haplotypes represent a combination of these three variants. For example, the C3435-G2667-C123 (CGC) haplotype is defined as the wild-type haplotype, whereas the 3435T-2677T-123T (TTT) is classified as the mutant haplotype. Therefore, CGC/CGC is proposed for individuals who are homozygous for the reference alleles from the three variants (i.e., C3435-G2667-C123/ C3435-G2667-C123). In contrast, TTT/TTT is anticipated for individuals with two minor alleles from the three *ABCB1* variants (i.e., 3435T-2677T-123T/3435T-2677T-123T).

With regards to the predicted paraoxonase activity for the p.Q192R (rs662) variant, individuals with the mutant-type RR genotype are anticipated to exhibit a high paraoxonase activity followed by those with the QR genotype display an intermediate enzymatic function while individuals with the wild type QQ genotype are expected to have the lowest level of paraoxonase activity. In contrast, for the p.L55M (rs854560) variant, serum paraoxonase is expected to be the highest with individuals carrying the reference LL genotype, followed by the individuals having intermediate serum concentration with the LM genotype, and the lowest for those having two minor alleles of the variant (i.e., MM genotype) due to reduction in the stability of the enzyme. The different haplotypes for *PON1* variants are based on the combination of the two p.L55M and p.Q192R genotype results.
